# Acknowledgement to reviewers

**DOI:** 10.1530/ETJ-15-A1

**Published:** 2026-02-05

The editorial board wishes to thank the following individuals who have helped to review manuscripts for the *European Thyroid Journal* during 2025. Their assistance is gratefully acknowledged.

T Akamizu

F Albarel

S L Andersen

G Åstrøm Ueland

B Asvold

C Badiu

M Bagnasco

M Barczyński

S Bárez-López

L Bartalena

A Bauer

T Bednarczuk

C Bellevicine

S Bernardi

A Bikas

K Boelaert

A Boelen

F Bogazzi

F Borson-Chazot

G Brigante

C Buffet

H B Burch

M Burlacu

B Cakir

M Cakir

J Calissendorff

I Campi

M Capezzone

P Caria

A Carle

P Caron

M G Castagna

A C Cavallo

F S Celi

S Censi

S Chanock

T Chiba

R Ciampi

V Cirello

R J Clifton-Bligh

C Colombo

V Condello

R P Coppes

R Corbo

B Corvilain

R Coutant

C Cunningham

T Cuny

D Danilovic

C Daumerie

C E Daumerie

C Dayan

S De Leo

B Decallonne

P Dedhia

K Demircan

M Dentice

F Di Fermo

L Difilippo

J M Dora

L Duntas

G Effraimidis

F Eilsberger

R Elisei

C Ellervik

M F Erdogan

M Ettleson

J Fei

C Ferraz

F Flamant

F Forrer

S Frara

A Frasoldati

G Fu

L Fugazzola

D Fuhrer-Sakel

T Fukuhara

J Galofré

I Ganly

E Giannetta

M Gild

A Glover

A R Glover

B Goichot

G Grani

E S Grassi

L Grixti

H Guan

A Gunnerbeck

M Gurnell

J Hadoux

C Han

D Handkiewicz-Junak

J Hang

M Haymart

A Heijboer

J V Hennessey

R Hoermann

K Horiuchi

P Hou

A Hubalewska-Dydejczyk

F Illouz

M Ito

Y Ito

D Janus

B Jarzab

M J Jeon

G J Kahaly

Z Kara

O Karapanou

W G Kim

A Kober

J Koehrle

P A Kopp

T Korevaar

J Krajewska

A Kus

A Kyriacou

L Lamartina

I Landa

G Lanzolla

H Lasolle

F Latrofa

K Latupeirissa

L J Leandro-Garcia

S Leboulleux

V Lee

A M Leung

Q Lian

J Lifante

X Lin

R Liu

W Liu

Z Liu

L Locati

M Lopez

C Lubitz

D Luton

X Lyu

E Macerola

F Magri

A L Maia

J Mammen

M Marino

V Marotta

A Matrone

G Mauri

S Mayerl

E Mcaninch

R M Melillo

F Menconi

F Menegaux

C Mian

K Minamitani

E Minna

A Mishra

N Mitsutake

C Modica

L Möller

H Monpeyssen

C Montero-Conde

C Mooij

J H Moon

C Moran

S Morelli

R Moreno-Reyes

S Moritani

L Morton

I Muller

E Nagy

M Nannini

B G Nedrebø

R Negro

R Netea-Maier

M Niedziela

Y E Nikiforov

A Nikitski

C Nucera

H F Nyström

K Ohba

O Okosieme

A Olivieri

R Opitz

T M Ortiga-Carvalho

J Osinga

F Pani

Y J Park

R Paschke

E N Pearce

S Pearce

G Pellegriti

P Perros

L Persani

M A Petkar

A Piccardo

A Piovesan

F Pitoia

T Planck

M Polak

K Poppe

K G Poppe

T Porcelli

N Pozdeyev

E Puxeddu

W Rankin

F Raue

S Razvi

S Refetoff

K Renko

J Reverter

J C Ricarte-Filho

A Ritter

P Rodien

C Romei

L Rossi

M Rotondi

G Russ

P Sadow

N Sakata

A Sanabria

J Sanders

N Satoshi

F Savagner

L Schomburg

X Shen

Y Shen

O Shidlovskyi

P Spegel

M Sponziello

M N Stan

L States

A Stoupa

K Sugino

I Sugitani

G Sykiotis

G Szinnai

G Tallini

E Talpacci

J Tan

P N Taylor

H Teng

W Teng

A Teumer

E Torlone

F Torresan

M Trevisan

P Trimboli

O Tsybrovskyy

J Tuckermann

C Ugolini

M Urken

B Vaidya

F Vaisman

A Van Den Bruel

D Van Heemst

A S Van Trotsenburg

G Vatine

F Verburg

W E Visser

J Wadsley

M Wasniewska

A Watanabe

N Watanabe

X Wei

J Wemeau

E K Wirth

L Wirth

C L Wood

J X Wu

Y Xu

H Yamazaki

V Yartsev

Y Yoshida

S Yu

M Zafereo

C Zafon

M Žarković

